# The development of medical MOOCs in China: current situation and challenges

**DOI:** 10.1080/10872981.2018.1527624

**Published:** 2018-10-04

**Authors:** Zhaohui Gong

**Affiliations:** Department of Biochemistry and Molecular Biology, Medical School of Ningbo University, Ningbo, Zhejiang, China

**Keywords:** Massive open online courses, medicine, higher education, certificate, impact

## Abstract

This study aimed to investigate the current situation and challenges on the development of medical massive open online courses (MOOCs) in China. A survey was constructed and the statistical analysis was adopted to evaluate the medical MOOCs. The results showed that the medical MOOC-related journal papers, conference papers, books and dissertations have risen dramatically over the past five years. In addition, the top 6 most representative MOOC platforms provide the majority (87%) of medical courses. The statistical analysis showed that PMPH-MOOC was the most influential medical MOOC platform in China. Compared to the foreign medical MOOCs, medical MOOCs in China were urgently in need of systemic planning, interaction activities and international certification. Overall, the findings suggest that the development of medical MOOCs for higher education has been achieved a great success along with a huge challenge.

## Introduction

Massive open online courses (MOOCs) are open-access online courses that allow for unlimited participation and have revolutionized universities to corporate education landscape [1]. In addition to traditional course materials such as filmed lectures and readings, MOOCs provide interactive user forums to support community interactions among participating students and teaching professors. The term MOOC was originally coined by George Siemens and Stephen Downes in fall 2008. They offered the first online course ‘Connectivism and Connective Knowledge’ for free and organized the course in a real-time manner in the University of Manitoba [2]. During the development of MOOCs, early MOOCs mainly stressed the open-access features, such as open licensing of content, structure and learning objectives, to promote the reuse of resources. Later MOOCs often use closed licenses for their course materials to provide certificates for students [3]. Learning outside the classroom through MOOCs is severely challenging the conventional education and is profoundly changing the individual learning styles [4,5].

From 2008, many colleges and universities around the world began to launch companies and curriculums that have the characteristics of MOOC. The digital and mobile technology are extremely needed in medical education [6,7]. In the era of healthcare, MOOCs are widely infiltrate into radiology learning [8], nursing education [9], ophthalmology education [10], pharmacy education [11], parasitology education [12], and even global health [13]. In China, the symbol of MOOC development was that ‘online school’, the first MOOC platform led by universities, was launched in 2013 [14]. Now the development of MOOC in China is extremely rapid, and is still growing. In the age of globalization, China’s accreditation of medical education is promoting the quality of medical education and talent training [15]. Medical MOOCs are deeply involved in higher education and are becoming important resources.

In our previous study, we demonstrated the most productive and influential biochemistry textbooks, editors and publishing houses, and revealed the usage status of biochemistry textbook in medicine [16]. Although MOOCs have made great progress in past years, the current situation of medical MOOCs in China and the different of medical MOOCs between inside China and outside China have not been reported at the global level for medical educators.

Here we took a complete and reliable survey to investigate the situation of medical MOOCs in China using the search tools provided by National Knowledge Infrastructure (CNKI) and National Library of China (NLC). By analysing the data, we found that journal papers, conference papers, books and PhD/master dissertations about medical MOOCs increased dramatically in the past five years. Six leading platform provides 87% medical MOOCs and MOOC (icourse163) is the largest course platform, while CNMMOC has the largest number of medical MOOCs in China. Moreover, different platforms have different percentage of medical courses and partner universities. Interestingly, the top 3 domestic platforms with medical MOOCs almost shared the same number of courses with the top 3 similar foreign platforms. Further comparison showed medical MOOCs in China are urgently in need of systemic planning, professional staffs, online and offline activities, and international certification. This work demonstrates that the growth and utilization of medical MOOCs in China higher education have been staggering. However, it also has to meet the challenges that lie ahead.

## Methods and analysis

### Study subjects and data collection

The publications, including journal papers, conference papers, books and PhD/master dissertations, were obtained from the CNKI (cnki.net) and NLC (nlc.gov.cn) websites using the keywords ‘MOCCs’ and ‘medical education’. The deadline was set for April 20^th^, 2018. Then the publication information, such as author, title, affiliation, publication year etc., was extracted and converted into an Excel document. We also selected the top 10 most representative domestic MOOC platforms and top 5 most representative foreign MOOC platforms to collect the platform provider, number of total and medical MOOCs, course information, instructor, and certificates. The completeness, accuracy and consistency of the collected data was confirmed by three different independent investigators.

### Classification and statistics of publications

Based on the publication types, the collected publications were divided into four different types: (1) Journal paper, (2) Conference paper, (3) Book and (4) PhD/master dissertation. The number of different publications was calculated by type and year (2014–2018). The data for year 2018 was analysed before April 20^th^.

### Survey on the status of medical moocs

By the end of April 20^th^, 2018, we investigated the top 10 most representative MOOC platforms inside of China and top 5 most representative MOOC platforms outside of China. The total courses and medical courses were analysed and the proportion of medical courses in total courses was also calculated and compared among these MOOC platforms. Since MOOC platforms were intelligently supported by many universities, the partner universities on each platform were also analysed. In addition, we compared the situation of medical MOOCs between inside of China and outside of China.

### Evaluation of the impact of medical MOOC platform

To evaluate the impact of medical MOOC platform in China, we introduced a new concept of ‘Impact Factor (IF)’. In detail, the value of the proportion of medical courses in total course was set as a direct IF (DIF) for this MOOC platform. In the past one year, the sum of participants in medical courses was calculated. Then, the average value of the number of medical courses divided by the sum of participants was set as an average IF (AIF). The total IF (TIF) equals to the sum of DIF and AIF. The impact of medical MOOC platform was ranked by the TIF value.

### Comparison of medical MOOC platforms between inside of china and outside of china

The top 3 most representative medical MOOC platforms that are inside of China and outside of China were respectively chosen for comparison. The differences in content structures, professional staffs, online/offline activities and international certificates were compared and listed in a table.

### Statistical analysis

The data analyses were performed using SPSS 13.0 software.

## Results

### The medical mooc-related publications have risen dramatically over the past five years

**Figure 1.**
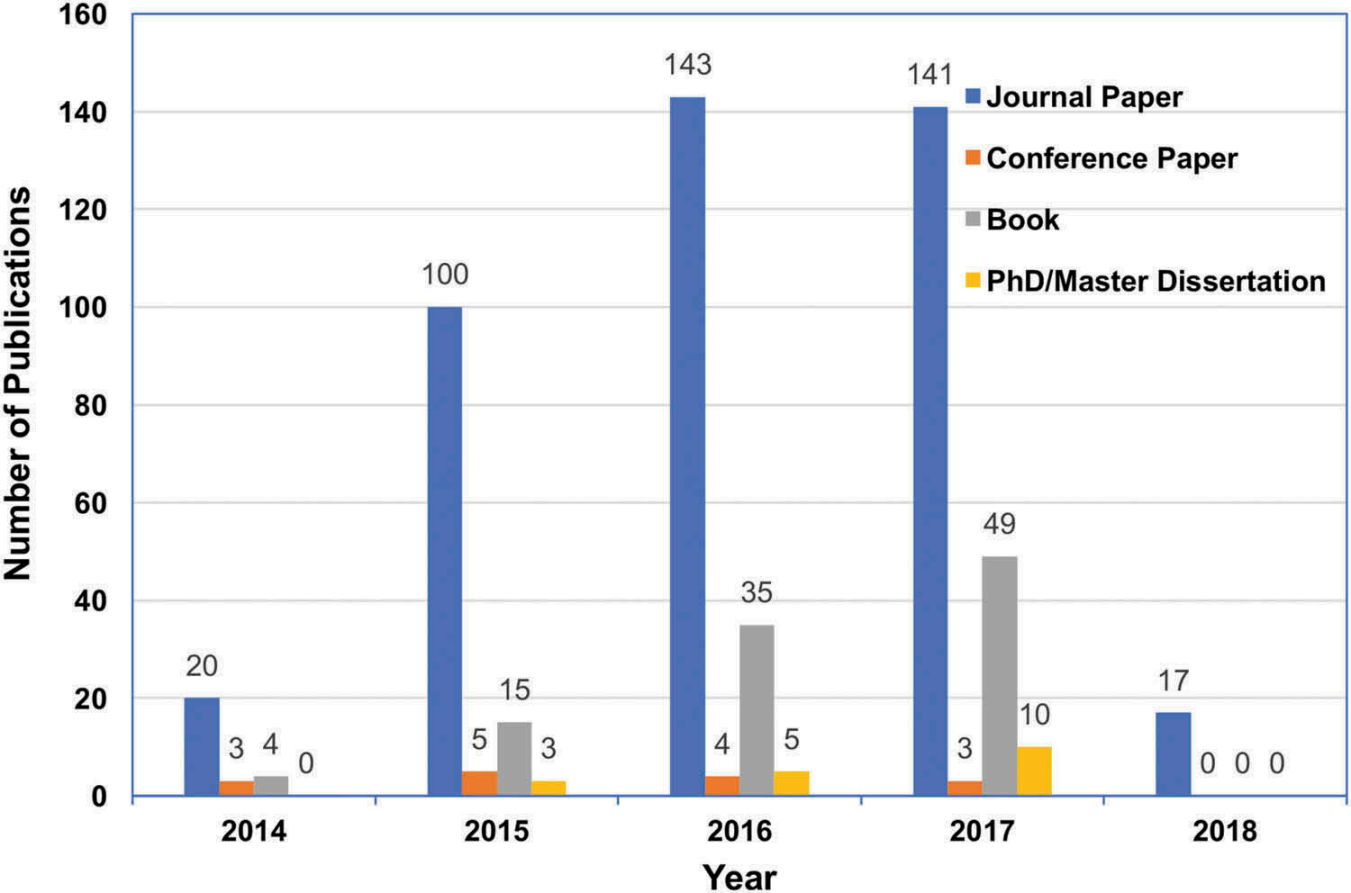
The change of the number of medical MOOC-related Chinese publications in the past 5 years.

CNKI is China’s largest web-based publication platform and it includes more than 7,000 journals, 1,000 newspapers, 180,000 PhD/master dissertations, 160,000 conference papers, etc. In addition, NLC has a collection of over 31.1 million items and is the largest library in Asia and one of the largest in the world. Using the online search tools provided by CNKI and NLC, we obtained the medical MOOC-related publications. From January 2014 to 20 April 2018, there were 421 journal papers, 15 conference papers, 103 books and 18 PhD/master dissertations published in the field of medical MOOCs. In the past five years, the number of journal papers increased rapidly. Especially, the number of journal papers in 2015 was five times of that in 2014. Similarly, the books and dissertations increased almost ten-fold in five years (Figure 1). Although the data for year 2018 was collected before April 20^th^, the growth trend of publication was still clear.

### Main MOOC platforms provide the majority of medical courses

**Figure 2.**
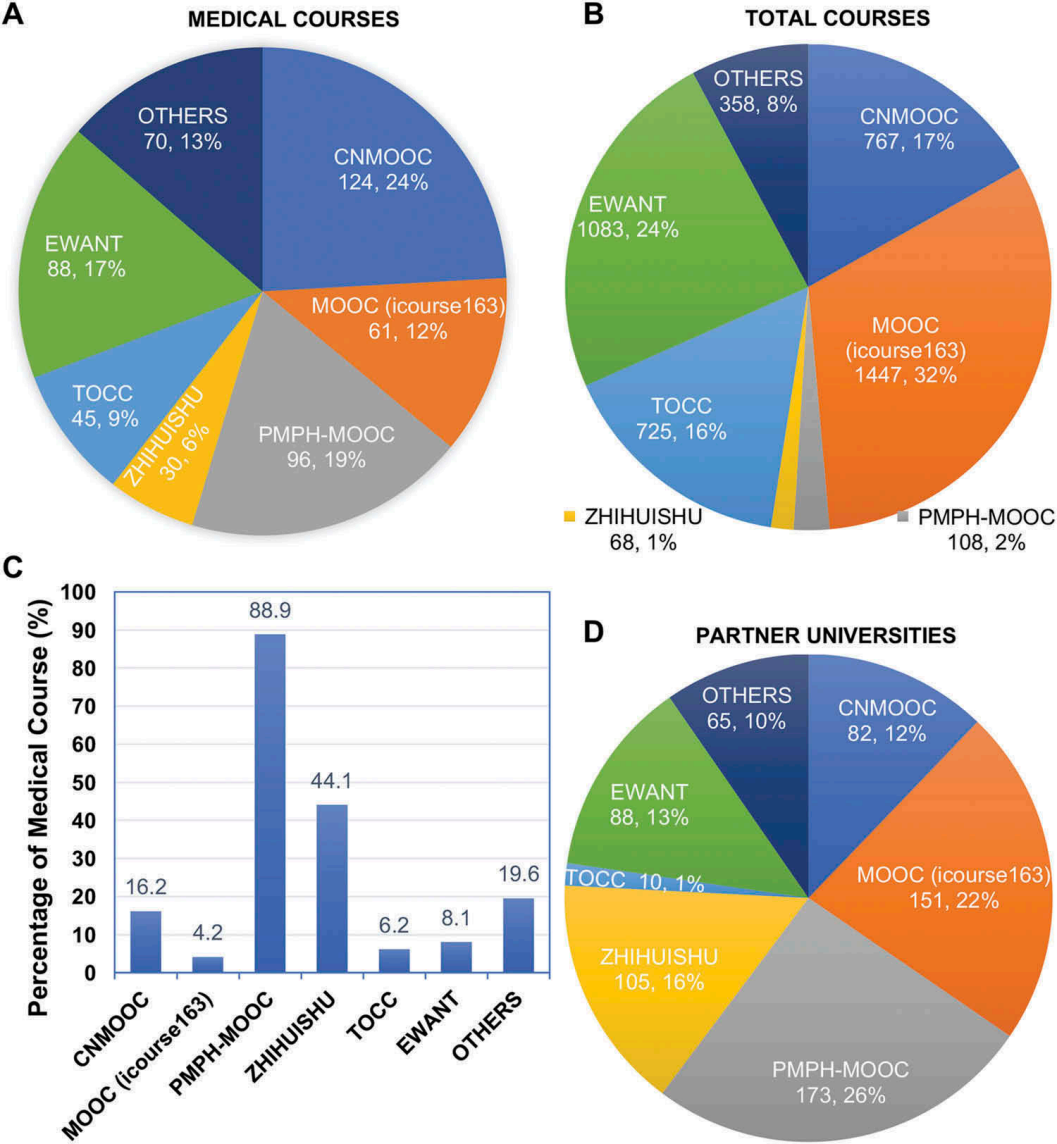
The current situation of medical MOOCs in different China MOOC platforms. (a) Medical Course, (b) Total courses, (c) Percentage of medical courses, and (D) Partner universities.

We compared the main Chinese MOOC platforms and ranked the platforms by the number of medical courses. The results showed that there were 440 medical courses on the top 6 most representative MOOC platforms, 70 medical courses on other platforms. The largest medical MOOC platform was CNMOOC (cnmooc.org) which held 124 medical courses (Figure 2A). For the total courses, there were 4,198 courses on the main MOOC platforms. The largest MOOC platform of MOOC (icourse163) (icourse163.org) had 1,447 courses in total (Figure 2B). Among these main platforms, different platforms provided different proportions of medical courses in total ones. The PMPH-MOOC platform (pmphmooc.com) had the highest proportion of medical course with 88.9% and the MOOC (icourse163) had the lowest one with 4.2% (Figure 2C). Moreover, the main MOOC platforms were intelligently supported by many universities. There were 674 partner universities participated in the development of medical MOOCs. Among these platforms, the PMPH-MOOC cooperated with 173 partner universities and yielded the highest percentage of 26% (Figure 2D). These results indicated that the number of the medical MOOCs increased rapidly in the past years and the top 6 most representative MOOC platforms provided more than 80% medical courses for higher education. The development of MOOCs has brought up some reputed medical MOOC platforms, such as PMPH-MOOC and ZHIHUISHU (zhihuishu.com).

### PMPH-MOOC is the most influential medical MOOC platform

To further investigate the impact of medical MOOC platform in China, we calculated the DIF and AIF, and then we evaluated the TIF for each MOOC platform. The results showed that CNMOOC had the highest DIF with 24 and ZHIHUISHU had the lowest DIF with 6. Meanwhile, PMPH-MOOC reached its highest AIF with 4875.3 and EWANT shared the lowest AIF with 78.4. Taken together, PMPH-MOOC yielded the maximum value (4894.3) for TIF and became the most influential medical MOOC platform in China (Table 1). Interestingly, although the DIF of ZHIHUISHU was the lowest one, it had a higher AIF value. Among these platforms, EWANT and TOCC were developed in Taiwan (a part of China) and the participants were mainly from Taiwan. If all Chinese people could freely access these two platforms, they would become more influential. It’s well known that the five MOOC platforms except for PMPH-MOOC are comprehensive and multidisciplinary platforms, and PMPH-MOOC was founded by people’s medical press house, the largest medical publisher in China, and mainly focused on the medical courses. Certainly, PMPH-MOOC will be the most influential medical MOOC platform for China medical education in the foreseeable future.

**Table 1.** The impact of medical MOOC platforms.

IF			
Pltform	DIF	AIF	TIF
CNMOOC	24	232.7	256.7
PMPH-MOOC	19	4875.3	4894.3
EWANT	17	78.4	95.4
MOOC(icourse163)	12	4496.1	4508.1
TOCC	9	125.7	134.7
ZHIHUISHU	6	3451.6	3457.6

IF, impact factor; DIF, direct impact factor; AIF, average impact factor; TIF, total impact factor.

### China medical moocs were processing rapidly with lack of innovations

**Figure 3.**
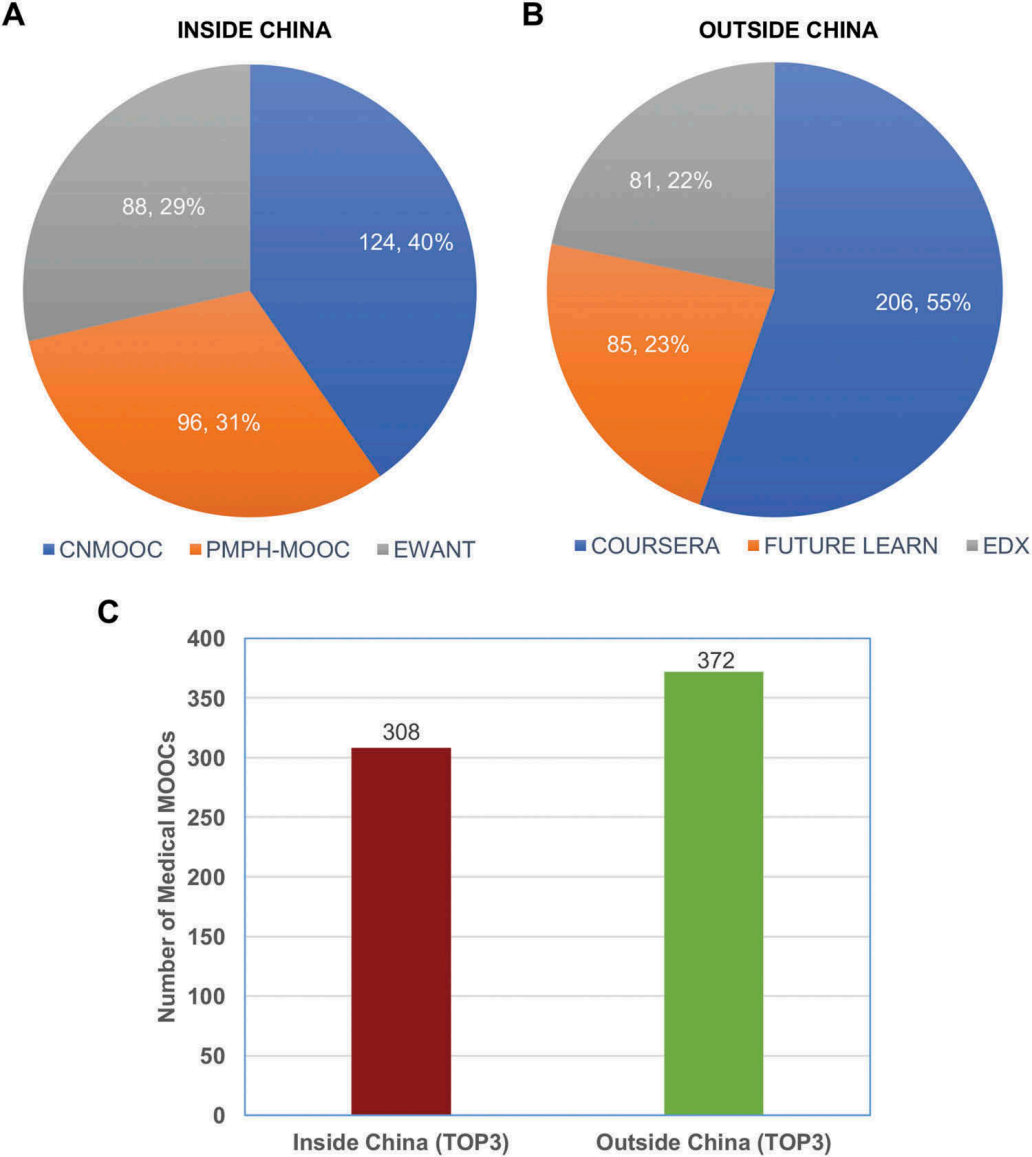
The comparison of medical courses from top 3 most representative MOOC platforms between inside and outside of China. (a) Inside of China, (b) Outside of China, and (c) Number of medical MOOCs.

To compare the situations of medical MOOCs between inside and outside of China, we selected top 3 most representative domestic and foreign medical MOOC platforms, respectively. The top 3 most representative domestic MOOC platforms shared 308 medical courses provided by CNMOOC, PMPH-MOOC and EWANT (Figure 3A). While the top 3 most representative foreign MOOC platforms shared 372 medical courses provided by COURSERA, FUTURE LEARN and EDX (Figure 3B). From total look, there was no significant difference in total medical courses between inside and outside of China (Figure 3C). The data indicated that China medical MOOCs had achieved similar progress with the foreign platforms.

However, the development of medical MOOCs inside of China was lack of innovation. Most medical MOOCs were migrated from previous web-based online courses, nearly half (48%) consisted of courseware and text information. Compared to outside of China, the medical MOOCs inside of China were insufficient of system plan. Different MOOC platforms were short of integrated design and shared many common repeated courses. In addition, most medical courses didn’t have specified audiences, such as medical students, continuing educator or general public. The interaction in Chinese medical MOOCs were insufficient. For example, some medical courses had no answer-question section and some questions were left with no replies. Statistically, there were more than half (51%) of student’s questions that were not answered. Although both inside and outside of China medical MOOCs had the similar high-level instructors and course resources, the quality of medical courses inside of China was not the same as that outside of China. Particularly, the video and audio quality need to be improved. Most importantly, the learning evaluation in Chinese medical MOOCs varied from that in foreign ones. Inside of China medical MOOCs were mainly evaluated in the manner of summative path, outside of China medical MOOCs were evaluated by the process assessment. Only less than 10% of medical MOOCs inside of China were awarded credits, while more than 50% of medical MOOCs outside of China were given credits. Moreover, most (> 80%) inside of China medical MOOCs issued unpaid certificates, only less than 15% inside of China medical MOOCs offered paid certificates and more than 50% outside of China medical MOOCs offered paid certificates (Table 2). The medical MOOCs with free certificates and no credits would affect the learning quality of students. Together, there was a big difference of medical MOOCs between inside and outside of China.

**Table 2.** The comparison of medical MOOCs between inside and outside of China.

	Inside of China	Outside of China
System plan	Fair	Good
Interaction	Insufficient	Sufficient
Instructor	Good	Good
Resource	Multimedia	Multimedia
Learning evaluation	Summative assessment	Process assessment
Credit	< 10%	> 50%
Certificate	Paid < 15%	Paid > 50%

## Conclusions and discussion

In this paper, a complete and reliable survey to investigate the situation of medical MOOCs in China was conducted and we found that the publications about medical MOOCs increased dramatically in the last five years. Top 6 most representative MOOC platforms provided most of the medical MOOCs and PMPH-MOOC was the most influential medical MOOC platform. Compared to the foreign medical MOOCs, medical MOOCs in China are urgently in need of systemic planning, interaction activities and international certification.

In the era of big data, MOOC has shown its own benefits in education [17]. More and more academic unions, institutions and commercial firms are providing a lot of medical MOOCs for higher education. Although there are many benefits to medical MOOCs, they cannot take place of the traditional medical education in hospital and colleges. And, for this, we should take advantage of its benefits and combine MOOC and traditional classroom closely, change the role of the teacher and student to improve the education quality of medical courses [18].

Nevertheless, the difference of medical MOOCs between inside and outside of China should not be ignored. In the meantime, there are still some challenges that needed to be overcome. For participants, a certain amount of digital literacy is a necessary condition for the use of network information. In addition, participants may need more time and effort than students are willing to commit to free online courses. Once the course is released, the content will be reshaped and interpreted by a large number of students. This makes it difficult for teachers to control the course. Some medical courses should be carried out in a real environment, it’s more difficult to reproduce reliably the contents offered by the virtual online materials [19]. Furthermore, participants must have strong sense of self-motivation, self-management and ability to work under pressure. The general challenges in effective medical MOOC development are usually accompanied by criticism by journalists and academics.
